# Supplementary material for: FDA-ARGOS is a database with public quality-controlled reference genomes for diagnostic use and regulatory science
Source: Nat Commun. 2019 Jul 25;10:3313. doi: 10.1038/s41467-019-11306-6 (PMC6658474; doi:10.1038/s41467-019-11306-6)
Supplement: Supplementary file 1 — Supplementary Information [file 41467_2019_11306_MOESM1_ESM.pdf]

Supplementary Information:

FDA-ARGOS: Public Quality-Controlled Reference Genomes for Diagnostic Use and Regulatory Science

Sichtig et. al.

Supplementary Table 1

A: FDA-ARGOS Metadata Coverage

Metadata #	Metadata Name	# Samples	% Samples
1	ORGANISM	487	100.00%
2	STRAIN	487	100.00%
3	IDENTIFICATION METHOD	487	100.00%
4	ISOLATION SOURCE	487	100.00%
5	HOST	487	100.00%
6	COLLECTED BY	487	100.00%
7	CULTURE COLLECTION	487	100.00%
8	TAXONOMY_ID	487	100.00%
9	CONTACT:EMAIL	487	100.00%
10	PACKAGE	487	100.00%
Addtl	geo_loc_name	400	82.14%
Addtl	collection_date	362	74.33%
Addtl	host_disease	303	62.22%
Addtl	host_sex	205	42.09%
Addtl	host_age	200	41.07%

B: FDA-ARGOS PACKAGE Metadata Coverage

PACKAGE	# Samples	% Samples
Clinical	399	81.93%
Environmental	88	18.07%

C: FDA-ARGOS IDENTIFICATION METHOD Metadata Coverage

IDENTIFICATION METHOD	# Samples	% Samples
phenotypic identification/ characterization	347	71.25%
16S and MLST	26	5.34%
RT-PCR and amplicon sequencing	23	4.72%
Phenotypic and Molecular Methods	22	4.52%
RT-PCR	22	4.52%
culture	21	4.31%
real-time PCR, EM, deep sequencing	9	1.85%
culture, 16S	5	1.03%
isoenzymes, DNA sequencing	2	0.41%
PCR, DNA sequencing	1	0.21%
culture and biochemical testing	1	0.21%
culture and phenotypic identification	1	0.21%
culture, culture and spot test, serum bactericidal assay	1	0.21%
culture, plasmid agarose gel electrophoresis, phenotypic identification	1	0.21%
culture, repetitive element PCR	1	0.21%
Gram stain and anaerobic culture	1	0.21%
histology of at least 2 biospy sites, Warthin-Starry silver stain	1	0.21%
MLST	1	0.21%
PCR - multiple targets	1	0.21%

Supplementary Table 2

A: Metagenomics E. Avium CLC Genomics Workbench Analysis (Total Mapped Reads)

Species	Replicate 1	Percent	Replicate 2	Percent	Replicate 3	Percent	Average	Percent
E. avium	379	0.0033	571	0.0045	1226	0.0107	725.33	0.01
H. sapiens	10996939	96.23	12020125	95.75	9434390	82.56	10817151.33	91.52

B: Metagenomics E. Avium CLC Genomics Workbench Analysis (Reads in Aligned Pairs)

Species	Replicate 1	Percent	Replicate 2	Percent	Replicate 3	Percent	Average	Percent
E. avium	362	0.0032	558	0.0044	1202	0.0105	424.40	0.01
H. sapiens	9695338	84.84	10374660	82.65	8082686	70.73	5630570.30	79.41

C: Metagenomics E. Avium CLC Genomics Workbench Analysis (Total Reads)

Replicate 1	Replicate 2	Replicate 3	Average
11427304	12553052	11427304	11802553.33

Supplementary Table 3

<u>Limit of Detection (LoD) Table EBOV Serum</u>

PFU/ml	Median EBOV Reads	Median Percent Reads Mapped	CoV	Min - Max Mapped Reads per Index	Positive Detection
100000 (run 1)	75107	0.067290926	0.932770546	1049-167863	20/20
100000 (run 2)	67330.5	0.066819427	0.934177256	1199-257990	20/20
NTC	33.5	0.004173915	-	7-107	-

Notes:

- Precision across the 40 replicates performed on two different sequencing runs on different days = 5.50%.
- Run 1 contained 28,663,203 and Run 2 contained 25,064,302 reads post trim.
