# Supplementary material for: FDA-ARGOS is a database with public quality-controlled reference genomes for diagnostic use and regulatory science
Source: Nat Commun. 2019 Jul 25;10:3313. doi: 10.1038/s41467-019-11306-6 (PMC6658474; doi:10.1038/s41467-019-11306-6)
Supplement: Supplementary file 11 — Reporting Summary [file 41467_2019_11306_MOESM11_ESM.pdf]

natureresearch

Corresponding author(s): Heike Sichtig, Timothy Minogue

Last updated by author(s): Jun 28, 2019

Reporting Summary

Nature Research wishes to improve the reproducibility of the work that we publish. This form provides structure for consistency and transparency in reporting. For further information on Nature Research policies, see Authors & Referees and the Editorial Policy Checklist.

Statistics

For all statistical analyses, confirm that the following items are present in the figure legend, table legend, main text, or Methods section.

n/a	Confirmed	
☐	☒	The exact sample size (*n*) for each experimental group/condition, given as a discrete number and unit of measurement
☐	☒	A statement on whether measurements were taken from distinct samples or whether the same sample was measured repeatedly
☐	☒	The statistical test(s) used AND whether they are one- or two-sided<br>*Only common tests should be described solely by name; describe more complex techniques in the Methods section.*
☐	☒	A description of all covariates tested
☐	☒	A description of any assumptions or corrections, such as tests of normality and adjustment for multiple comparisons
☐	☒	A full description of the statistical parameters including central tendency (e.g. means) or other basic estimates (e.g. regression coefficient) AND variation (e.g. standard deviation) or associated estimates of uncertainty (e.g. confidence intervals)
☐	☒	For null hypothesis testing, the test statistic (e.g. *F*, *t*, *r*) with confidence intervals, effect sizes, degrees of freedom and *P* value noted<br>*Give P values as exact values whenever suitable.*
☐	☒	For Bayesian analysis, information on the choice of priors and Markov chain Monte Carlo settings
☒	☐	For hierarchical and complex designs, identification of the appropriate level for tests and full reporting of outcomes
☐	☒	Estimates of effect sizes (e.g. Cohen's *d*, Pearson's *r*), indicating how they were calculated

Our web collection on statistics for biologists contains articles on many of the points above.

Software and code

Policy information about availability of computer code

Data collection	no software was used
Data analysis	The MegaBLAST function of blast+ 2.7.1 installed on FDA HPC infrastructure (https://www.ncbi.nlm.nih.gov/books/NBK153387/) was used to taxonomically classify the short reads using the default parameters and the 200 database instance assembly sets. Each of the 200 database instance assembly sets was made into a nucleotide database using the makeblastdb command. For this study, the taxon associated with the first reported alignment sorted by max alignment score was used as the taxonomic label for each read. Original MegaBLAST results were summarized to report the number of reads associated with each unique NCBI taxonomy ID called.<br><br>Kraken 1.0, installed on FDA HPC infrastructure (https://ccb.jhu.edu/software/kraken/MANUAL.html), was used to assign a taxonomic label to each short read using default parameters and the same 200 database instance assembly sets. The database instance assembly sets were built into Kraken databases using the default options. Original Kraken results were summarized to report the number of reads associated with each unique NCBI taxonomy ID called.<br><br>LMAT version 1.2.6 (available for download at sourceforge.net/lmat, (Ames et al., 2015)), installed on Lawrence Livermore National Laboratory (LLNL) HPC infrastructure was used to assign a taxonomic label to each short read with a minimum score setting of 0.5. Match scores are calculated per read, by fitting a random null model created by simulating 1 GB of random sequence for each model dependent on read length and GC content. Three databases, the Algorithm Standard Database (LMAT DB), the stand-alone FDA-ARGOS and an aggregated database consisting of both the LMAT DB database and the stand-alone FDA-ARGOS database were used. LMAT results were summarized to report the number of reads associated with each unique NCBI taxonomy ID.

For manuscripts utilizing custom algorithms or software that are central to the research but not yet described in published literature, software must be made available to editors/reviewers. We strongly encourage code deposition in a community repository (e.g. GitHub). See the Nature Research guidelines for submitting code & software for further information.

Data

Policy information about availability of data

All manuscripts must include a data availability statement. This statement should provide the following information, where applicable:

- Accession codes, unique identifiers, or web links for publicly available datasets
- A list of figures that have associated raw data
- A description of any restrictions on data availability

• All FDA-ARGOS reference genome raw data, assemblies, annotations, metadata, base modification data and pipeline information are available from Bioproject ID# PRJNA231221 and at https://www.ncbi.nlm.nih.gov/bioproject/231221.

• Supplementary Data 1: FDA-ARGOS Reference Genome Database: NCBI Accessions and Assembly Quality Metrics (487 novel FDA-ARGOS nucleotide sequences presented in this manuscript and deposited in the NCBI GenBank nucleotide database).

• Supplementary Data 2: Normalized NCBI Nt and FDA-ARGOS database instances (accession codes for all 200 database instance assembly sets from use case 1).

• Supplementary Data 3: Read Classification Results from Metagenomics Shotgun Data of Mock Clinical Human Blood Sample Spiked with 10^5 Enterococcus avium (Supplementary Data 3a: MegaBLAST Metagenome Raw Data, Supplementary Data 3b: Kraken Metagenome Raw Data).

• Supplementary Data 4: Read Classification Results from Isolate Shotgun Data of Spiked Enterococcus avium (Supplementary Data 4a: MegaBLAST Isolate Raw Data, Supplementary Data 4b: Kraken Isolate Raw Data).

• Supplementary Data 5: Benchmark and In Silico Performance of MIPS BDBV and EBOV Assay

• 5 Reference Datasets from the use cases are available from Bioproject ID# PRJNA495928 and at https://www.ncbi.nlm.nih.gov/bioproject/495928.
a. Metagenomic Shotgun Sequencing for Identification of E avium (3 replicate samples)
b. Isolate Shotgun Sequencing for Identification of E avium (3 replicate samples)
c .MIPS for Identification of Bundibugyo Virus (10 PCR positive, 1 NTC)
d. MIPS for Identification of Ebola Virus Makona (15 PCR positive, 1 NTC)
e. MIPS EBOV Mock Clinical Trial (148 blinded samples: 48 positive: 16 10X, 5X, 1X and 100 matrix-only negative)

Field-specific reporting

Please select the one below that is the best fit for your research. If you are not sure, read the appropriate sections before making your selection.

☒ Life sciences ☐ Behavioural & social sciences ☐ Ecological, evolutionary & environmental sciences

For a reference copy of the document with all sections, see nature.com/documents/nr-reporting-summary-flat.pdf

Life sciences study design

All studies must disclose on these points even when the disclosure is negative.

Sample size	Use Case 1: Three replicate mock clinical E. avium samples for metagenomic shotgun sequencing and three replicate cultured E.avium samples for isolate shotgun sequencing were selected for this use case. Triplicate samples were multiplexed to demonstrate feasibility and reproducibility of generating clinically relevant E. avium reads from metagenomic and isolate sequencing.<br><br>Use Case 2: Initial testing was performed using 10 clinical Bundibugyo ebolavirus PCR positive samples and 15 clinical Zaire ebolavirus Makona PCR positive samples. The sample sizes were selected based on availability at the testing site. We note that these type of samples are extremely hard to acquire. Comparison of the PCR positive data to ID-NGS test genome data and FDA-ARGOS reference genome data suggested a complete reliance on the inherent sensitivity of the ID-NGS assay for in silico sequence comparison. To document the application of MIPS EBOV ID-NGS assay benchmarking, we performed a mock clinical trial to assess the assay-specific wet-lab subset evaluation as part of the proposed novel composite reference method (C-RM). We selected standard mock clinical validation representing 100 negatives, 16 low, 16 medium and 16 high concentration samples. At a minimum, 15 samples were needed to provide a measure of statistical robustness to allow claims of positive predictive value (PPV) and negative predictive value (NPV).
Data exclusions	None
Replication	Replicate samples were run for all studies.
Randomization	Use case 2: 148 mock clinical samples were randomized.
Blinding	Use case 2: 10 clinical Bundibugyo and 15 clinical Ebola virus (EBOV) Makona samples were de-identified. 148 mock clinical samples were blinded.

Reporting for specific materials, systems and methods

We require information from authors about some types of materials, experimental systems and methods used in many studies. Here, indicate whether each material, system or method listed is relevant to your study. If you are not sure if a list item applies to your research, read the appropriate section before selecting a response.

Materials & experimental systems

n/a	Involved in the study
☒	☐ Antibodies
☒	☐ Eukaryotic cell lines
☒	☐ Palaeontology
☒	☐ Animals and other organisms
☐	☒ Human research participants
☒	☐ Clinical data

Methods

n/a	Involved in the study
☒	☐ ChIP-seq
☒	☐ Flow cytometry
☒	☐ MRI-based neuroimaging

Human research participants

Policy information about studies involving human research participants

Population characteristics	Fifteen de-identified human serum samples that were Ebola virus (EBOV) Makona positive were received from Sierra Leone; these samples were determined by the USAMRIID Office of Human Use and Ethics to be Not Human Subject Research (HP-09-32). Ten de-identified human serum samples that were suspected Bundibugyo virus positive were received from the Democratic Republic of Congo (DRC). These samples were determined by the USAMRIID Office of Human Use and Ethics to be Not Human Subject Research (HP-12-15).
Recruitment	See above.
Ethics oversight	USAMRIID Office of Human Use and Ethics

Note that full information on the approval of the study protocol must also be provided in the manuscript.
