# Supplementary material for: FDA-ARGOS is a database with public quality-controlled reference genomes for diagnostic use and regulatory science
Source: Nat Commun. 2019 Jul 25;10:3313. doi: 10.1038/s41467-019-11306-6 (PMC6658474; doi:10.1038/s41467-019-11306-6)
Supplement: Supplementary file 12 — Description of Additional Supplementary Files [file 41467_2019_11306_MOESM12_ESM.pdf]

- Title: Supplementary Data 1
  Description: FDA-ARGOS Reference Genome Database: NCBI Accessions and Assembly Quality Metrics (487 novel FDA-ARGOS nucleotide sequences presented in this manuscript and deposited in the NCBI GenBank nucleotide database).

  Title: Supplementary Data 2
  Description: Normalized NCBI Nt and FDA-ARGOS database instances (accession codes for all 200 database instance assembly sets from use case 1).

  Title: Supplementary Data 3
  Description: Read Classification Results from Metagenomics Shotgun Data of Mock Clinical Human Blood Sample Spiked with 10^5 *Enterococcus avium*

  Title: Supplementary Data 4
  Description: MegaBLAST *E.avium* Metagenome Raw Data

  Title: Supplementary Data 5
  Description: Kraken *E.avium* Metagenome Raw Data

  Title: Supplementary Data 6
  Description: Read Classification Results from Isolate Shotgun Data of Spiked *Enterococcus avium*

  Title: Supplementary Data 7
  Description: MegaBLAST *E.avium* Isolate Raw Data

  Title: Supplementary Data 8
  Description: Kraken *E.avium* Isolate Raw Data

  Title: Supplementary Data 9
  Description: Benchmark and *In Silico* Performance of MIPS BDBV and EBOV Assay
